# Cellular Nutrition in Complex Three-Dimensional Scaffolds: A Comparison between Experiments and Computer Simulations

**DOI:** 10.1155/2015/584362

**Published:** 2015-10-11

**Authors:** Claudia Bergemann, Patrick Elter, Regina Lange, Volker Weißmann, Harald Hansmann, Ernst-Dieter Klinkenberg, Barbara Nebe

**Affiliations:** ^1^Department of Cell Biology, University Medical Center Rostock, Schillingallee 69, 18057 Rostock, Germany; ^2^University of Applied Sciences Mittelhessen, Wiesenstraße 14, 35390 Giessen, Germany; ^3^Faculty of Computer Science and Electrical Engineering, Institute for Electronic Appliances and Circuits, University of Rostock, Albert-Einstein-Straße 2, 18059 Rostock, Germany; ^4^Institute for Polymer Technology, Alter Holzhafen 19, 23966 Wismar, Germany; ^5^DOT GmbH, Charles-Darwin-Ring 1a, 18059 Rostock, Germany

## Abstract

Studies on bone cell ingrowth into synthetic, porous three-dimensional (3D) implants showed difficulties arising from impaired cellular proliferation and differentiation in the core region of these scaffolds with increasing scaffold volume *in vitro*. Therefore, we developed an *in vitro* perfusion cell culture module, which allows the analysis of cells in the interior of scaffolds under different medium flow rates. For each flow rate the cell viability was measured and compared with results from computer simulations that predict the local oxygen supply and shear stress inside the scaffold based on the finite element method. We found that the local cell viability correlates with the local oxygen concentration and the local shear stress. On the one hand the oxygen supply of the cells in the core becomes optimal with a higher perfusion flow. On the other hand shear stress caused by high flow rates impedes cell vitality, especially at the surface of the scaffold. Our results demonstrate that both parameters must be considered to derive an optimal nutrient flow rate.

## 1. Introduction

In current therapeutic strategies, large bone defects caused by trauma, tumors, or infections are filled by bone auto- or allografts [1, 2]. These methods imply disadvantages such as limited availability, donor site morbidities, immunological reactions, or the risk of infections [3–5]. Synthetic implants provide an alternative to the limited resources of autografts and the problems in the use of allogenic or xenogenic grafts. The success of such implants is determined by various factors: the materials used have to be biocompatible and corrosion-resistant, they must have the correct mechanical properties, and the architecture of the graft has to favor tissue ingrowth into the scaffold.

Commonly, synthetic three-dimensional (3D) scaffolds were used, whose structures were phenomenologically optimized for cell seeding [6–8]. However,* in vitro* studies of bone cell ingrowth into scaffolds demonstrated an impaired cellular proliferation and reduced differentiation in the core region of scaffolds with increasing scaffold volume [9, 10]. Consequently, osteoblast growth into porous scaffolds with pore sizes between 400 *μ*m and 800 *μ*m in diameter [11, 12] was observed, but migration to the interior of such scaffolds was limited for static* in vitro* cultures without nutrient flow [13]. The results were interpreted by a concentration gradient from the surface to the core due to a restriction of medium diffusion in the scaffold, followed by insufficient nutrient and oxygen supply (hypoxia) and waste accumulation (acidification) for cells in the core region [9, 14, 15]. Hypoxia influences osteogenic differentiation in cell cultures [16–18] and may cause cell death inside the implant [10]. Therefore, cell nutrition in the core region of a scaffold is frequently supported by medium flow* in vitro*, with perfusion bioreactor systems yielding the best results [19–23].

Commercial perfusion systems for tissue culture, for example, Minucells perfusion containers (MINUCELLS and MINUTISSUE Vertriebs GmbH, Bad Abbach, Germany), the OsteoGen bioreactor (Instron, Norwood, USA), the 3D Bioreactor (3D Biotek, Sigma-Aldrich, Munich, Germany), or the Z^*®*^RP Bioreactor (Zellwerk, Oberkrämer, Germany) as well as custom-made bioreactors, were used in published studies [19–25]. However, the use of dynamic cell cultures reveals a dilemma: while sufficient cell nutrition requires relatively high medium flow rates, the faster movement of the liquid leads to increased shear stress and as a consequence to increased levels of cell death [26–28]. The local supply rates as well as the local shear stress depend on the exact channel structure inside the scaffold and, consequently, an optimal medium flow rate has to be determined for each scaffold architecture. In turn, this means that not every channel structure allows an optimal supply for the cells.

To gain a better understanding of the nutrient supply in the scaffold, the experiments were supported by computer simulations [29–34]. The applied methods range from Brownian Dynamics Simulations that explicitly calculate the trajectory of each nutrient particle [31] to simulations on finite element basis [34]. Frequently, the diffusion and the directed flow of a particle species are taken into account and the local balance of consumed and freshly supplied molecules is calculated [34]. In particular, many studies focus on the simulation of the oxygen concentration inside the scaffold and come to the result that the oxygen supply is a critical factor in determining cell fate [32–34]. However, the calculated channel structures [31, 32] are very simple and allow only limited conclusions for real systems.

To overcome this limitation, an* in vitro* 3D cell culture module was developed that allows the cultivation of osteoblasts in a 3D porous structure at different nutrient flow rates. The system was especially designed to allow cell analysis in the scaffold interior. We compared the wet-lab data (cell viability) with those from computer simulations. These* in silico* data based on the finite element method (FEM) predicted the local oxygen supply and shear stress inside the scaffold and let us draw conclusions for the optimization of perfusion flow rates and the channel design of the scaffold.

## 2. Material and Methods

### 2.1. *In Vitro* 3D Module

#### 2.1.1. Tantalum (Ta) Scaffold and Clamping Ring

**Figure 1:**
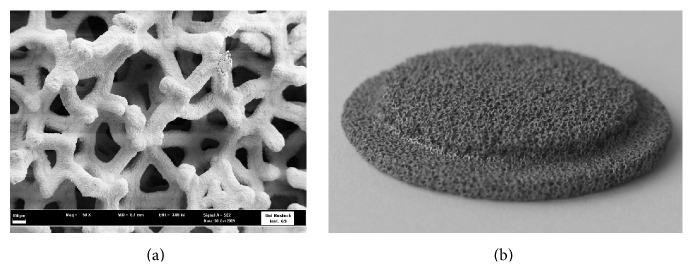
(a) Scanning electron microscopic (FESEM) image showing the pore structure of the Ta scaffold (magnification 50x, bar 100 *μ*m). (b) Image of the molded Ta scaffold (radius: 14 mm, center height: 5 mm).

**Figure 2:**
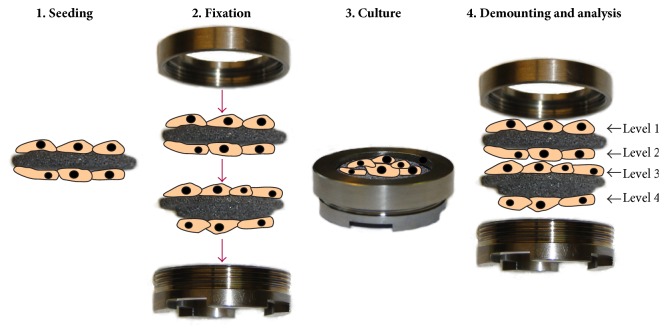
Schematic view of the horizontal fixation of two Ta scaffolds in the clamping ring forming the* in vitro* 3D module with four separate levels.

Ta scaffolds (Zimmer, Freiburg, Germany) of 14 mm radius and 5 mm thickness were used (Figure 1). This porous trabecular Ta has a typical porosity of 80% and a pore size of around 550 *μ*m. The scaffolds were molded on one side at the edge to fit into the clamping ring (Figure 2). The Ta scaffolds and the clamping ring were sterilized by autoclaving (121°C steam, Superior, Mammooth, Italy). The clamping ring was made of titanium (grade 2) with an inner radius of 12 mm, and a mounting thread ensured that the plane surfaces were situated on top of each other. In this way the* in vitro* 3D module simulated one scaffold (total height: 10 mm), enabling nondestructive cell observation on four different levels without cutting the material: one apical (level 1), two medial (levels 2 and 3), and one basal (level 4) surface.

#### 2.1.2. Cell Seeding for the 3D Module

MG-63 osteoblastic cells (osteosarcoma cell line, ATCC, LGC Promochem, Wesel, Germany) were used as a well-established cell model for* in vitro* research in biomaterials science [33–36]. Cells were cultured in Dulbecco's modified Eagle medium (DMEM) (Invitrogen, Darmstadt, Germany) supplemented with 10% fetal calf serum (FCS) (PAA Gold, PAA Laboratories, Cölbe, Germany) and 1% gentamicin (Ratiopharm, Ulm, Germany) at 37°C in a humidified atmosphere with 5% CO_2_. Near confluence, cells were detached with 0.05% trypsin/0.02% EDTA for 5 min. After stopping trypsinization by the addition of cell culture medium, an aliquot of 100 *μ*L was put into 10 mL of CASY ton buffer solution (Roche Innovatis, Reutlingen, Germany) and the cell number was measured in the counter CASY Model DT (Schärfe System, Reutlingen, Germany).

Two porous 3D Ta scaffolds with the molded side up were placed into a 6-well tissue culture plate (Greiner Bio One, Frickenhausen, Germany) and covered with 4 mL of DMEM. 1 · 10^6^ cells in 100 *μ*L DMEM were seeded in a meandering pattern onto the surface of the medium-incubated Ta scaffolds. After 15 min, when the cells had adhered to the materials, the 6-well plate was incubated at 37°C in a humidified atmosphere with 5% CO_2_ for another 6 h. Then the Ta scaffolds were inverted (molded side down) and the seeding procedure was repeated for the opposite sides. After a further incubation overnight at 37°C in a humidified atmosphere with 5% CO_2_, the two porous 3D Ta scaffolds were stacked horizontally and fixed within the clamping ring (Figure 2), with the plane surfaces on each other.

### 2.2. Perfusion Cell Culture Reactor

**Figure 3:**
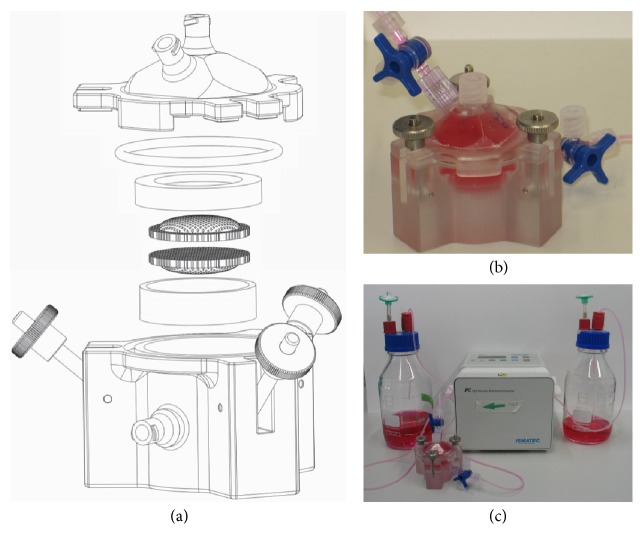
(a) Schematic view and (b) image of the perfusion cell culture reactor “Cellynyzer” with the integrated* in vitro* 3D module for dynamic cell culture of large scaffolds followed by nondestructive cell analysis. (c) Equipment of the* in vitro* 3D module: connections to the medium and waste reservoirs and to the peristaltic pump.

For 3D dynamic cell cultures in scaffolds, we developed not only the* in vitro* 3D module but also a perfusion cell culture reactor (Cellynyzer, Institute for Polymer Technologies Wismar, Germany) [35]. This cell culture reactor was made by rapid prototyping on the basis of a biocompatible methacrylate resin (FotoMed LED.A, Innovation MediTech GmbH, Germany) (height of 60 mm and 25 mm in radius). The interior was cylindrical, designed to precisely fit the 3D module to guarantee perfusion, and completed by three Luer cones for the connection to Luer Lock systems (Figure 3). The reactor was conceived to be extendable in height by adding a spacer ring between the base and the upper section. More than two scaffolds or heightened scaffolds could thereby be easily incorporated into the system.

The perfusion cell culture reactor “Cellynyzer” was sterilized by autoclaving (121°C steam, Mammooth). Gas permeable tubes (Fluidflex Silicon HG, LIQUID-scan, Germany) were chosen for the supply of medium to the cell culture reactor due to their pH maintenance at 37°C in a humidified atmosphere with 5% CO_2_. The tubes were sterilized by rinsing with ethanol (70%), sterile PBS, and cell culture medium before connecting them to the cell culture reactor. The cell reactor was connected to a peristaltic pump (IPC-N 4, Ismatec, Germany), a medium reservoir, and a waste container. The pump and the medium reservoir were placed outside the incubator: the pump at room temperature and the medium in the refrigerator standing beside it. The medium was heated to 37°C by elongating the tube inside the incubator up to 50 cm before reaching the reactor. The pump was calibrated before the experiments and medium consumption was measured during the experiments.

### 2.3. Dynamic Cell Culture

The seeded* in vitro* 3D module (see Figure 2) was integrated into the perfusion cell culture reactor. The cell culture medium was pumped through the 3D module with a flow direction being bottom-up within the reactor. Dynamic culture experiments were done in complete DMEM as described for 7 days under continuous flow with different flow rates (5, 15, 30, 45, and 60 *μ*L/min). Control experiments were done under static culture conditions without flow in the same cell reactor. For this purpose, the culture medium was removed using a sterile syringe and carefully replaced by new medium every other day. All static and dynamic experiments were done twice. After cell culturing for the appropriate time under the defined medium flow, cells on every level of the* in vitro* 3D module underwent a separate cell-biological analysis. This was easily realized by demounting the module and taking the two scaffolds apart (see Figure 2). In this way, cells from the core of the* in vitro* 3D module could be analyzed without cutting any material and cell observation was therefore nondestructive.

### 2.4. Nondestructive Cell Analysis

#### 2.4.1. Cell Vitality

MG-63 cells were cultured in the* in vitro* 3D module system in dynamic as well as static mode (without flow) for 7 days as described. Vitality was analyzed by live/dead staining (L7013 LIVE/DEAD Cell Viability Kit, Invitrogen, Germany) after demounting the Ta scaffold, washing carefully with Hank's Buffered Salt Solution (HBSS, PAA Laboratories GmbH, Germany) and staining the cells with a solution of SYTO 10 and DEAD Red (8 *μ*L of each dye in 4 mL HBSS) for 15 min at 37°C in the dark. Then the scaffolds were inverted and incubated for another 15 min at 37°C in the dark; the scaffolds were washed with HBSS and incubated with 4% glutaraldehyde in HBSS for at least 1 h at 4°C. Cells were examined on the fluorescence microscope (Axio Scope.A1, 10x air lens, Carl Zeiss, Germany) using the standard fluorescein long pass filter. The nuclei of living cells appeared green and could thereby be distinguished from dead cells (red nuclei).

#### 2.4.2. Field Emission Scanning Electron Microscopy (FESEM)

The porous structure of the Ta scaffold material was examined by FESEM (SUPRA 25, Zeiss, Germany). Cell density on the different levels of the* in vitro* 3D module was visualized after 7 days of culture under static conditions. For this purpose, the upper and lower Ta scaffolds were separated (see Figure 2), washed with PBS, fixed with 4% glutaraldehyde, and dehydrated using a graded series of acetone. After critical point drying (K 850, EMITECH, Germany), the scaffolds were examined by FESEM.

### 2.5. Computer Simulations

#### 2.5.1. Computer Model

**Figure 4:**
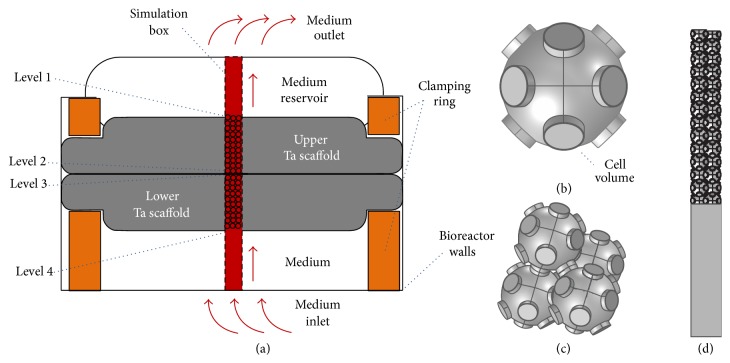
Computer model of the cell population in the 3D channel structure of the Ta scaffold. (a) Cross section of the perfusion reactor with the two cell-populated Ta scaffolds inside. The simulation box is located in the center of the figure. The porous channel structure of the scaffold is modeled by interconnected spherical cavities whose walls are occupied by cells. (b) Double-walled cavity with connections to adjacent cavities. The narrow region between the inner and the outer wall represents the cell-populated area. (c) Construction of the channel structure from many cavities. (d) Lower part of the simulation box consists of the cross section though the Ta disk and the medium reservoir are below the disk. Periodic boundary conditions are assumed (see text for details).

The oxygen supply of the cell culture was analyzed by a computer model based on the finite element method (FEM). The perfusion reactor and the simulation box are illustrated in Figure 4(a). The simulation box consisted of a section through the structured scaffold as well as the reservoir regions below and above the Ta scaffold. For reasons of computing time the simulation box was split into a lower region with the medium inlet and an upper part with the medium outlet; both regions were linked together by the equating of the concentrations and pressures at their contact faces.

The channel structure of the Ta scaffold was modeled by double-walled connected spheres (Figures 4(b)–4(d)); the narrow region between the outer and inner wall represents the volume in which the cells were located. Periodic boundary conditions were assumed in the horizontal directions (Figure 4(d)); each end face was linked to the symmetry-equivalent end face on the opposite site of the simulation box leading to a total number of 94 periodic boundary conditions for each half of the simulation box. However, along the main flow direction of the medium (bottom-up) the full scaffold disc was calculated.

The local oxygen concentration was evaluated for a dense cell layer on the channel walls of the scaffold. It was derived from the balance of consumed molecules by the cells and supplied molecules by diffusion and by inflowing medium. The external reservoir of fresh medium was assumed to be large compared to the volume of the perfusion reactor, and consequently the oxygen concentration was set to a constant bulk value of *c*_0_ outside the reactor as well as at the inlet-/outlet-interfaces. In the cell-occupied regions oxygen is consumed at a given consumption rate *R*.

For the calculation of the oxygen concentration, the simulation procedures of Coletti et al. [33] and Buchwald et al. [34] were adapted to the current geometry. The diffusion of oxygen was described by the general diffusion equation [34]

(1) $$\begin{array}{c} \frac{\partial c}{\partial t}+\nabla \cdot \left(\;-D\nabla c\;\right)=R-u\cdot \nabla c, \end{array}$$

where *c* is the local oxygen concentration, *D* is the diffusion constant of oxygen in the medium, ∇ is the Nabla-Operator, and **u** is the local velocity vector of the media. Because the oxygen consumption of the cells depends on the locally available amount of oxygen, a Michaelis-Menten-type expression was used for *R*, similar to the calculations of Buchwald et al. [34]:

(2) $$\begin{array}{c} R=\left\{\begin{array}{cc} 0, & \text{culture medium,}\\ R_{max}\cdot \frac{c}{c+c_{MM}}\cdot \delta \left(c\geq c_{c}\right), & \text{cell volume.} \end{array}\right. \end{array}$$

In the equation *R*_max_ is the maximal oxygen consumption rate of the osteoblast cells, *c*_MM_, the Michaelis-Menten constant, that is, the oxygen concentration at which the cells consume only 50% of *R*_max_. *c*_*c*_ is a critical oxygen concentration at which the cells die (and then consume no more oxygen). The delta function *δ* returns a value of 1 for *c* ≥ *c*_*c*_ and a value of 0 otherwise. It ensures that oxygen consumption ceases (*R* = 0) for regions with dead cells (*c* < *c*_*c*_). The local velocity field **u** in (1) was calculated by solving the Navier-Stokes equation [33, 34]

(3) $$\begin{array}{c} \rho \frac{\partial u}{\partial t}+\rho \left(\;u\cdot \nabla \;\right)u+\nabla p=\eta \cdot \nabla^{2}u+f \end{array}$$

for an incompressible liquid medium (∇·**u** = 0). Here, *ρ* is the density of the liquid medium, *η* is the viscosity, and **f** is the vector of the body force density.

#### 2.5.2. Simulation Parameters

The oxygen concentration *c*_0_ for a fresh medium at 37°C was set to 0.42 mol/m^3^; the diffusion constant *D* of oxygen was set to 2.68 · 10^−9^ m^2^/s [36]. For reasons of comparability, the values for the critical concentration and the Michaelis-Menten constant were taken from the simulations of Buchwald et al. [34] and set to *c*_*c*_ = 10^−4^ mol/m^3^ and *c*_MM_ = 10^−3^ mol/m^3^. Oxygen consumption rates vary widely in the literature. For example, Wang et al. [37] demonstrated that the consumption rates of osteoblasts vary between 5.56 · 10^−6^ *μ*mol/(cell·min) for osteoblasts cultured in static T-flasks and 1.25 · 10^−7^ *μ*mol/(cell·min) for encapsulated medium* in vitro*. Considering the cell numbers and the cell volume given in [37], oxygen consumption rates between *R*_max_ = 2.7 · 10^−2^ mol/(m^3^·s) and *R*_max_ = 1.23 · 10^−4^ mol/(m^3^·s) arise. Accordingly, an average oxygen consumption rate of *R*_max_ = 2.0 · 10^−3^ mol/(m^3^·s) was used in this study.

Medium supply rates of 5, 15, 30, 45, and 60 *μ*L/min as well as the static cell culture were analyzed. Please note that all specified flow rates refer to the entire scaffold (such as in the experiments) and are internally converted to the region of the simulation box by analyzing the ratio of the respective surfaces. A surface of 4.523 · 10^−4^ m^2^ was used for the scaffold (representing the inner region of the Ta disk, which is not blocked by the clamping ring) and 8.40 · 10^−7^ m^2^ for the simulation box.

The porous channel structure of the scaffold was constructed by double-walled spheres with an outer radius of 250 *μ*m and an inner radius of 245 *μ*m (thickness of the cell layer: 5 *μ*m). The connections were built up of double-walled cylinders with an outer radius of 80 *μ*m, an inner radius of 75 *μ*m, and a connection length of 2 · 25 *μ*m. The channel structure was meshed with a free tetrahedral mesh (1,244,382 elements) and the equations were solved using the parallel direct sparse solver (PARDISO). The iteration was terminated when the relative error was less than 10^−3^. A finer mesh or a larger number of iterations did not yield relevant improvements in test simulations. All calculations were performed with COMSOL Multiphysics version 4.2a and carried out on an Intel Xeon X5680 processor (2 processors with 3.33 GHz and 48 GB of main memory).

## 3. Results and Discussion

### 3.1. Dynamic Cell Culture in the* In Vitro* 3D Module

Perfusion culture in a bioreactor with a pump system that perfuses nutrition media directly through the cell-seeded scaffold can obviously mitigate internal diffusional limitations which result in cell death [23, 38]. Different perfusion bioreactor systems used with different flow rates or velocities are mentioned in the literature [19–23]. Perfusion ensures an adequate supply to the cells but higher continuous flow rates led to increased levels of cell death due to shear stress [26–28]. Optimal flow velocities depend on the scaffold volume and the pore size and have to be determined separately for each perfusion system. In earlier studies we could demonstrate colonization of Ta scaffolds by MG-63 osteoblastic cells in our* in vitro* 3D module [39, 40]. To investigate cell survival inside scaffolds with dimensions suitable for large bone defects, the same module was used here. A setup in which all levels of the 3D module were seeded by osteoblast cells before assembling the module enabled the analysis of the cell survival in the core of the scaffold (see Figure 2). To promote the sufficient supply of the cells in the core of the scaffold we complemented the* in vitro* 3D module with a perfusion bioreactor, thus creating a novel dynamic system. Because the commercially available perfusion systems did not meet our demands for the size and special shape of our 3D module, we developed a custom-made direct perfusion bioreactor usable in a standard lab incubator with a humidified atmosphere and 5% CO_2_ [35].

**Figure 5:**
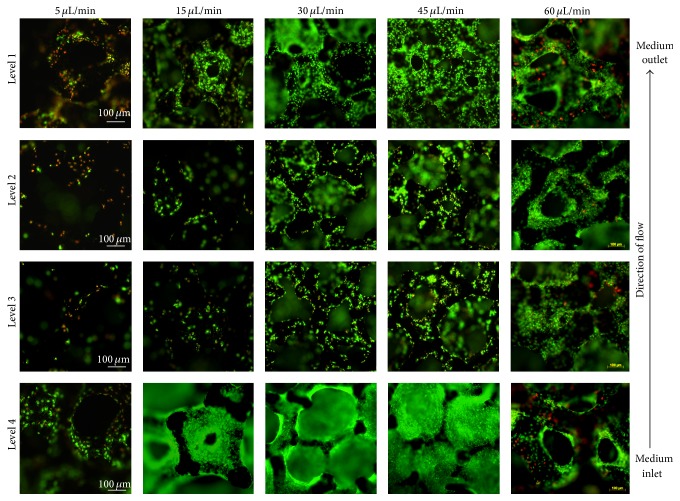
Dynamic cell culture with different medium flow rates for 7 days. Fluorescence images of MG-63 cell nuclei from the 4 different levels of the* in vitro* 3D module. Note that the best results for cell survival in the core region (levels 2 and 3) can be observed at perfusion flow rates of 30–45 *μ*L/min. Interestingly, cell death could be detected at a higher flow rate of 60 *μ*L/min, especially on level 4. Fluorescence images: nuclei of vital cells = green, nuclei of dead cells = red (Axio Scope.A1, magnification 10x, bar 100 *μ*m).

To identify the optimal flow rate for our system, we started with a very low pump velocity (5 *μ*L/min). Cell viability and cell density were analyzed on the different levels after 7 days of culture and demounting the scaffolds from the dynamic* in vitro* 3D module. As can be seen in Figure 5, insufficient cell survival occurred on all levels. Therefore velocity was enhanced progressively for the next experiments taking into consideration enhanced shear stress caused by high medium flow. Cell vitality on the different scaffold levels was correlated with the medium flow rate for every experiment.

In this way we found that cell survival rose on level 4 with a higher medium flow up to 45 *μ*L/min. Cell vitality and density in the core region (levels 2 and 3) were very low with flow rates below 30 *μ*L/min but increased at rates up to 60 *μ*L/min. As the flow direction in the cell culture reactor was bottom-up, cells on level 4 were always supplied with fresh medium. If the flow was too low, for example, 5 *μ*L/min, cells on the upper levels were supplied with used medium which was depleted of oxygen and nutrients and enriched with metabolic products by the cells from the levels below. Therefore it is not surprising that on levels 2-3 dead cells were detected at low flow rates. Interestingly, cell viability also decreased with the flow rate 60 *μ*L/min. Dead cells could be detected especially on level 4 and the cell density was reduced. This is an obvious contrast to the vitality on this level at 45 *μ*L/min. The higher continuous flow (60 *μ*L/min) seems to induce cell death. On the other hand, cell survival improved in the core (levels 2 and 3), but on these levels dead cells could be detected too. Therefore a flow rate of 60 *μ*L/min was considered as suboptimal.

**Figure 6:**
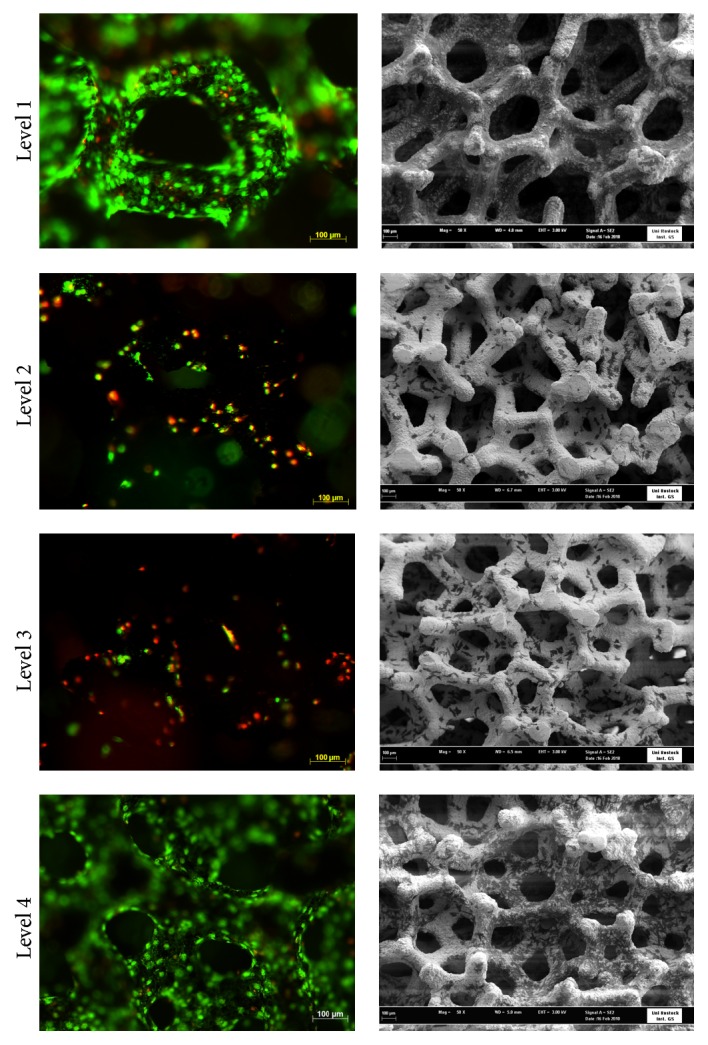
Static cell culture in the* in vitro* 3D module. Fluorescence (left) and FESEM images (right) of MG-63 cells after culturing for 7 days. Lower cell density and dead cells could be detected in the core region (levels 2 and 3). Fluorescence images: nuclei of vital cells = green, nuclei of dead cells = red (Axio Scope.A1, magnification 10x, bar 100 *μ*m) and FESEM images: Ta = white, cells = black (FESEM SUPRA 25, magnification 50x, bar 100 *μ*m).

Experiments with the same setup were done for 7 days without flow. Results of these static controls obtained high cell population in levels 1 and 4. Cells occupied the whole surface as could be seen in the FESEM images and were almost all vital on these levels (Figure 6). Cell survival in the core region (levels 2 and 3) was reduced as expected.

### 3.2. Simulation of the Oxygen Supply and Shear Stress

**Figure 7:**
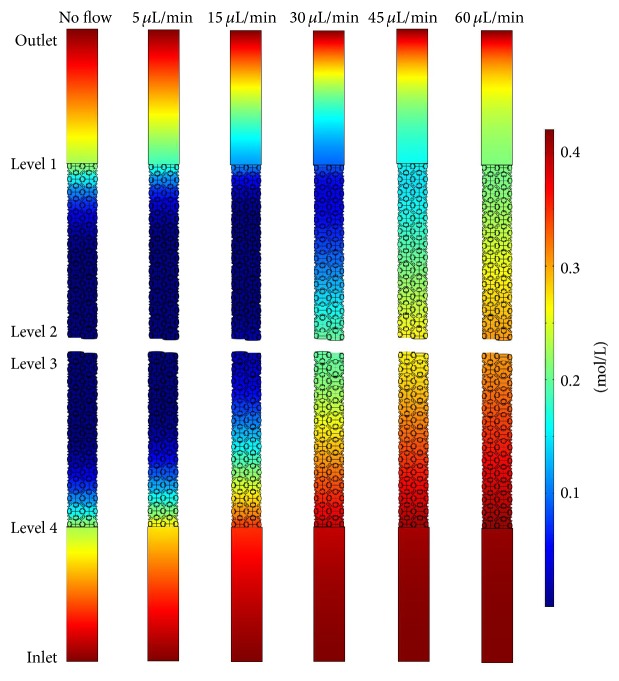
Local oxygen concentration in the simulation for different nutrient supply rates. The oxygen distribution shown is the balance between oxygen consumption in the cell-populated regions and oxygen supply by inflowing medium and by diffusion from the medium reservoirs.

To understand the nutrient supply in the scaffold, the experiments were supported by computer simulations.  Figure 7 displays the local oxygen concentration in the simulation box for different nutrient supply rates. The calculations are based on a dense cell colonization of the scaffold walls. The flow direction of the nutrient medium was from the bottom (inlet) to the top (outlet). Regions marked in red represent areas that are well supplied with oxygen, in yellow and green regions in which the oxygen concentration is significantly reduced, and in the dark blue areas a critical lack of oxygen.

Without flow the lowest oxygen concentrations were found in the middle of the scaffold, because new oxygen could diffuse from the inlet and from the outlet into the system but did not reach the inner regions before it was consumed. At low flow rates (5 *μ*L/min–30 *μ*L/min), the oxygen supply did not significantly improve, but the undersupplied region shifted in the direction of the outlet. The reason for this shift was that on the one hand fresh medium flowed into the system and increased the oxygen concentration in the vicinity of the inlet. On the other hand, however, the flow counteracted the inward diffusion of oxygen at the outlet so that the equilibrium concentration was reduced there. The system was only sufficiently supplied with oxygen at high flow rates (45 *μ*L/min–60 *μ*L/min), because incoming molecules were not completely consumed before they reached the region in the middle of the scaffold.

**Figure 8:**
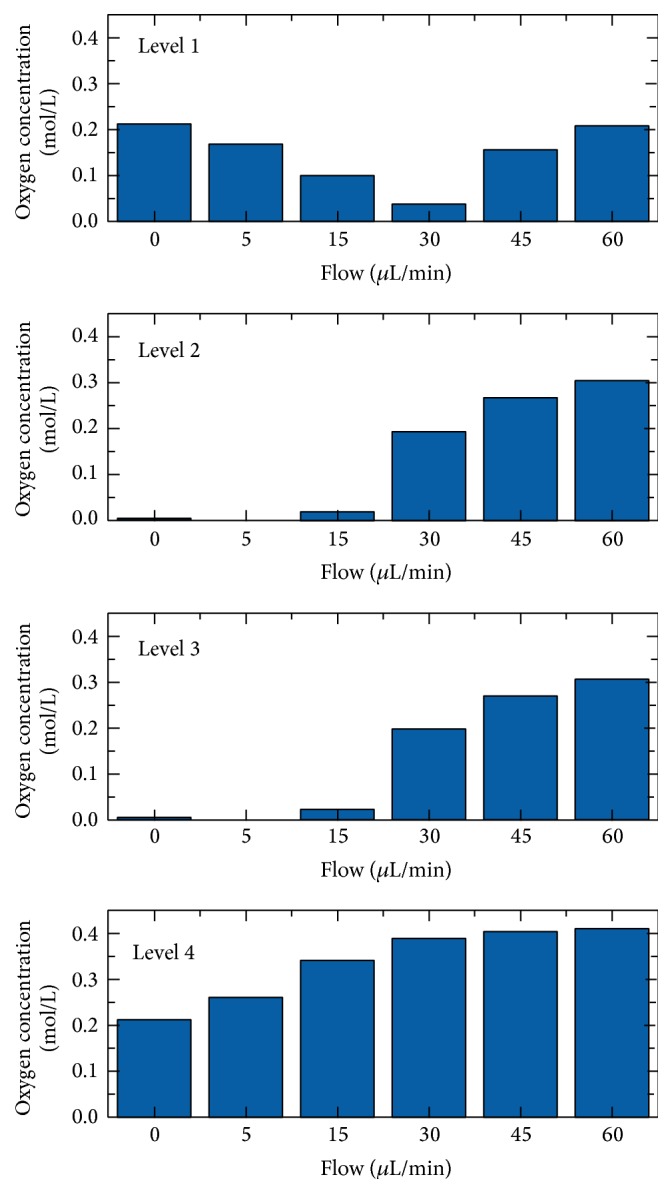
Oxygen concentration for the analysis levels 1–4 at different nutrient supply rates.

Figure 8 shows the oxygen concentration at the four analysis levels of the* in vitro* 3D module in the perfusion reactor for different flow rates. In the static cell culture, the oxygen concentration was reduced at level 1 (outlet) and level 4 (inlet) compared to fresh medium and levels 2 and 3 (in the core of the scaffold) were insufficiently supplied with oxygen. In comparison, the oxygen concentration decreased at level 1 and increased at level 4 for low flow rates (5 *μ*L/min–30 *μ*L/min); at level 2 and level 3 no significant change in the oxygen concentration was registered. Only at high flow rates (45 *μ*L/min–60 *μ*L/min) all levels were adequately supplied with oxygen.

**Figure 9:**
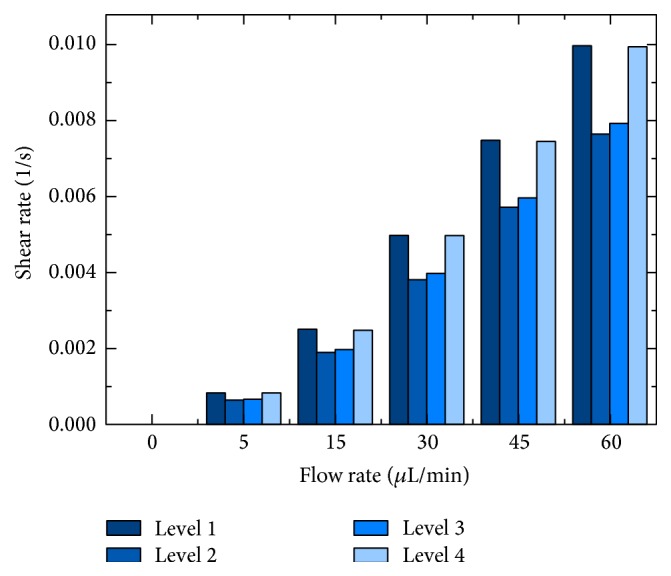
Shear rate for the analysis levels 1–4 at different nutrient supply rates.

In addition to the described changes in the oxygen concentration, increased shear rates have to be considered if the medium flow is increased.  Figure 9 displays the calculated shear rates at the cell surface for the four analysis levels and the different flow rates. The higher the flow rate, the greater the shear rate. However, the difference between the outer levels (1, 4) and the inner levels (2, 3) also increased at higher flow rates. These different shear rates may have an influence on the growth rate of the cells.

The comparison of results from the cell culture experiments in the dynamic 3D* in vitro* cell culture module and the computer simulation of oxygen supply and shear stress indicate a correlation between local population of vital cells and oxygen supply in the scaffold. By enhancing the medium flow insufficiently, supplied areas shift from levels 2 and 3 to level 4. Unfortunately, medium flow rates for adequate oxygen supply in all levels result in high levels of shear stress in the outer levels 1 and 4. Therefore it can be deduced that if oxygen supply does not impede the cell population in the scaffold, shear stress will. For lower medium flow rates between 15 *μ*L/min and 30 *μ*L/min cells will occupy the outer levels 1 and 4. With rising flow rates up to 45 *μ*L/min the cell vitality in the core region (levels 2 and 3) will grow through sufficient oxygen supply to the cells. For further enhanced flows up to 60 *μ*L/min the cell population will remain viable in the interior only (levels 2 and 3). Therefore a flow of 45 *μ*L/min can be assumed as the optimal medium flow rate for this dynamic 3D* in vitro* cell culture module.

However, please note that only the local balance of oxygen was considered in this study due to the high computation time of the complex 3D scaffold. Even if the presence of a sufficient amount of oxygen is believed to be a key factor for cell growth in tissue engineering [33], the presence of other molecules like nutrients and metabolic waste products may also influence cell fate. Nevertheless, Jonitz et al. were able to show a significantly reduced oxygen supply of cells in the core region of a similar 3D module already after one day of static culture, but no significant acidification by cell metabolites [41]. These results indicate that the local oxygen concentration is affected more quickly by the cells than other parameters and therefore is probably the most critical parameter for cell growth. Moreover, we would expect similar (e.g., for the nutrient molecules) or inverted (e.g., for metabolic products) concentration profiles as calculated for oxygen, because the diffusion and flow properties of these molecules do not fundamentally differ from oxygen. Consequently, the consideration of further molecules would moderately intensify or reduce the effects observed here but would not fundamentally change our interpretation.

In the present study, an* in vitro* 3D module with a perfusion cell culture reactor for dynamic cell cultures was developed and used to achieve enhanced cell viability in the core of scaffolds with dimensions suitable for large bone defects. The optimal flow rate for the survival of MG-63 cells was obtained at around 45 *μ*L/min in the dynamic mode, where fully cell-seeded scaffolds showed vital cells and cell death could not be detected on all four levels after 7 days of culture. Reduced cell survival with higher continuous flow rates could be attributed to cell death induced by shear stress, as shown by our simulation and as other groups also observed [26–28].

However, fluid shear stress was reported to induce osteoblast cell differentiation, whereby cell proliferation decreases [26, 28]. Sikavitsas et al. analyzed the influence of shear forces on rat bone marrow stromal cells with perfusing culture media of different viscosities and detected that mechanical stimulation enhanced the expression of the osteoblastic phenotype [28]. Cartmell et al. investigated the influence of enhanced medium flow on the cell proliferation and differentiation of MC3T3-E1 osteoblast-like cells perfused for 1 week at flow rates of 10, 100, and 200 *μ*L/min and 1.0 mL/min [26]. They showed enhanced cell proliferation at a flow rate of 10 *μ*L/min and induced cell differentiation at 200 *μ*L/min in bone constructs seeded with mouse osteoblast precursor cells. Campos et al. also determined cell differentiation dependent on fluid flow rates in a perfusion culture [24]. On hydroxyapatite/collagen scaffolds colonized with STRO-1A cells they found stimulated proliferation at flow rates of 300 *μ*L/min, but osteogenic marker gene expression was enhanced with a low flow of only 30 *μ*L/min. Therefore, the results seem to be contradictory and it is difficult to deduce the optimal flow from these examples. Taking into account that scaffold and pore size influence the distribution of the fluid and alter the shear forces generated in the scaffold, large scaffolds with higher pore size should require higher flow rates in perfusion culture than small ones. Li et al. [42] investigated the effects of flow shear stress on the construction of a large-scale tissue-engineered *β*-tricalcium phosphate scaffold (14 mm in diameter, 30 mm in height, with a tunnel of 3.5 mm in diameter) seeded with human bone marrow stromal cells. They used flow rates of 3.0 to 9.0 mL/min and altered shear forces by enhancing the viscosity of the medium. The results showed accelerated osteoblastic differentiation due to shear stress, while lower flow enhanced cell proliferation. A bidirectional continuous perfusion bioreactor for culturing constructs of significant dimensions was developed by Gardel et al. [43]. Fiber mesh scaffolds made from starch and polycaprolactone (16 mm in diameter, 3 mm in height) were seeded with goat marrow stromal cells and stacked, creating a 42 mm thick construct. The samples were cultured at a flow rate of 1.0 mL/min for up to 21 days. The number of cells in the constructs showed lower values compared to static culture, while dynamic conditions tended to enhance osteogenic differentiation. Cartmell et al. [26] also used scaffolds with higher volumes (6.35 mm in diameter, 6.35 mm in height) suitable for large bone defects. They were able to determine cell death in the constructs with an average pore size of 645 *μ*m at a flow rate of 1.0 mL/min and enhanced cell proliferation at 10 *μ*L/min. As insufficient cell supply in the core of a scaffold could result in impaired cell migration towards the center, peripherally seeded scaffolds should be cultured under optimal perfusion flow for cell survival in the core first, followed by induction of osteogenic differentiation through enhanced flow.

In this context, the optimal flow rate of 45 *μ*L/min for osteoblast survival in the combined large scaffold with a height of 10 mm in our dynamic* in vitro* 3D module was within the assumed range. In this preliminary study, we observed acceptable cell survival on the different levels after a cultivation time of seven days by live/dead staining of the cells. These results were confirmed by a simulation model for oxygen supply and shear stress. Using the optimal conditions for cell survival, our dynamic* in vitro* module can also be used for osteoblast migration studies by changing the cell seeding setup. For this purpose, cells could be seeded on only one outer level in the 3D module. In this way, active cell migration against gravity could be analyzed, for instance, dependent on the surface properties (chemical gradients) of the scaffolds. The optimal perfusion flow for osteogenic differentiation in our dynamic* in vitro* 3D module will be determined in further studies with mesenchymal stem cells or preosteoblasts. For optimal oxygen supply of all cells in the scaffold new architecture of scaffolds should be developed, where small cell-seeded channels are supplied with nutrients by diffusion from separate channels which are not seeded by cells. In these nonseeded channels higher medium flow rates would be possible.

## 4. Conclusions

The dynamic* in vitro* 3D module described here is a system well suited for the nondestructive investigation of cell behavior inside scaffolds with dimensions corresponding to large bone defects. For this 3D module, simulation of oxygen supply and shear stress combined with* in vitro* experiments result in an optimal flow rate near 45 *μ*L/min as a reasonable compromise. The 3D module combined with a dynamic cell culture in the perfusion reactor can be used for the observation of cell growth inside porous implant materials. Scaffolds with pore sizes of around 500 *μ*m and a minimum height of 10 mm can be investigated with respect to material composition and chemical surface modifications under an optimal perfusion flow rate.
